# Fibroblast Growth Factor 8 Deficiency Compromises the Functional Response of the Serotonergic System to Stress

**DOI:** 10.1371/journal.pone.0101420

**Published:** 2014-07-03

**Authors:** Leah R. Brooks, Heide L. Pals, Courtney L. Enix, Rachel A. Woolaver, Evan D. Paul, Christopher A. Lowry, Pei-San Tsai

**Affiliations:** Integrative Physiology and Center for Neuroscience, University of Colorado Boulder, Boulder, Colorado, United States of America; Radboud University, Netherlands

## Abstract

Functionally heterogeneous populations of serotonergic neurons, located within the dorsal raphe nucleus (DR), play a role in stress-related behaviors and neuropsychiatric illnesses such as anxiety and depression. Abnormal development of these neurons may permanently alter their structure and connections, making the organism more susceptible to anxiety-related disorders. A factor that critically regulates the development of serotonergic neurons is fibroblast growth factor 8 (Fgf8). In this study, we used acute restraint stress followed by behavioral testing to examine whether Fgf8 signaling during development is important for establishing functional stress- and anxiety-related DR neurocircuits in adulthood. Wild-type and heterozygous male mice globally hypomorphic for *Fgf8* were exposed to acute restraint stress and then tested for anxiety-like behavior on the elevated plus-maze. Further, we measured c-Fos immunostaining as a marker of serotonergic neuronal activation and tissue 5-hydroxyindoleacetic acid concentrations as a marker of serotonin functional output. Results showed that Fgf8 hypomorphs exhibited 1) an exaggerated response of DR anxiety-promoting circuits and 2) a blunted response of a DR panic-inhibiting circuit to stress, effects that together were associated with increased baseline anxiety-like behavior. Overall, our results provide a neural substrate upon which *Fgf8* deficiency could affect stress response and support the hypothesis that developmental disruptions of serotonergic neurons affect their postnatal functional integrity.

## Introduction

Many psychopathologies, including anxiety and affective disorders, may be neurodevelopmental in origin [1]. The serotonergic system is a key component of the anxiety circuitries. Ample evidence suggests environmental stressors can affect the serotonergic system to precipitate anxiety or affective disorders [2]–[5]. A conspicuous gap in our knowledge is how the abnormal development of serotonergic neurons may permanently alter their structure and connections to affect emotional behaviors. In this regard, it is important to investigate the actions of factors critical for the formation of the serotonergic system, since deficiencies in these factors can contribute to permanent and serious neurochemical and behavioral consequences.

The serotonergic system, spanning from the midbrain to the medulla, consists of functionally heterogeneous neurons that project to diverse forebrain and brainstem targets. The dorsal population of serotonergic neurons, or the dorsal raphe nucleus (DR), is located in the caudal midbrain and rostral pons [6]. The DR is further subdivided into dorsal (DRD), ventral (DRV), ventrolateral DR/ventrolateral periaqueductal gray (DRVL/VLPAG), interfascicular (DRI), and caudal (DRC) regions. These regions are functionally and topographically organized and project to regions responsible for modulating emotional and stress-related behavior, such as limbic and basal ganglia structures [7]. DRD serotonergic neurons project to forebrain regions that respond to anxiogenic drugs and stressors, such as the prelimbic (PrL) and infralimbic (IL) cortices, and the basolateral amygdala (BLA) [8], [9], suggesting a role in modulating anxiety-related circuitries. Further, surgical and pharmacological manipulations of the DR result in altered anxiety states [10]–[12]. There is also evidence that serotonergic neurons in the DRVL/VLPAG provide inhibitory input to the dorsal periaqueductal gray (DPAG) to attenuate panic-like responses to mild to moderate stressors [13], [14]. Therefore, developmental disruption of subpopulations of “anxiogenic” or “panic-inhibiting” serotonergic neurons and the associated connectivity may profoundly impact stress-related anxiety- and panic-like responses.

Fibroblast growth factors (Fgfs) and their receptors (Fgfrs) are critical for DR development [15]. *Fgf8* is expressed prenatally in the developing anterior hindbrain where DR serotonergic neurons arise, and secreted Fgf8 peptide forms a diffusion gradient that overlaps this region [15]. Deficient Fgf signaling, in particular Fgf8 and Fgf receptor (Fgfr) 1, results in fate specification failure and a loss of serotonergic neurons during development [16]–[21]. Fgf8 is not found in the postnatal DR [22]. Importantly, we recently showed that Fgf8 deficiency is associated with a loss of specific DR serotonergic neurons in the mid- to caudal DRV, DRVL/VLPAG, and DRI subregions and increased anxiety-like behavior [23]. However, it is unclear if the function and connectivity of anxiety- and panic-related DR subregions are also disrupted in these mice.

In this study, we tested the functionality of the developmentally compromised DR in Fgf8-deficient mice using acute restraint stress followed by behavioral testing. The goal of this study is to extend our previous findings by examining if Fgf8 deficiency disrupts the activation of DR serotonergic neurons, their functional output to anxiety- and panic-related projection regions, and anxiety-like behavior following stress [23]. Indeed, we found dysregulated responses to stress in both anxiety-promoting and panic-inhibiting circuits of Fgf8-deficient mice, which were associated with increased baseline anxiety-like behavior. Together these data expand our knowledge on the developmental factors needed to establish functional serotonergic circuits and related behavioral responses. Further, they raise the possibility that humans harboring *FGF8* loss-of-function mutations may exhibit abnormal anxiety- and stress-related responses [24].

## Materials and Methods

### Animals

All experiments were conducted using 8–10 week-old male wild-type (WT) or *Fgf8* heterozygous (HET) hypomorphic mice (129p2/OlaHsd* CD-1; obtained from Mouse Regional Resource Centers) [21]. *Fgf8* hypomorphic mice contain a neomycin-resistance element inserted into the non-coding region of the *Fgf8* gene. This element contains false splice sites that lead to about a 55% reduction in functional *Fgf8* transcript levels under homozygous condition [21]. *Fgf8* homozygous hypomorphic mice die within 24 h of birth but Fgf8 HET mice survive normally and have no obvious health problems. WT and Fgf8 HET mice were housed in same-sex littermate groups of 2–5 at weaning and genotyped by polymerase chain reaction of genomic DNA isolated from tail clips. All mice were bred at the University of Colorado Boulder in the Integrative Physiology department animal facility under a 12L: 12D photoperiod with free access to water and rodent chow. Prior to experiments, mice were left undisturbed except during routine cage changes. Experimental mice were moved to an adjacent room immediately prior to testing.

### Ethics statement

All animal procedures complied with the protocols approved by the Institutional Animal Care and Use Committee at the University of Colorado Boulder (Protocol # 1106.05) and adhered to the recommendations in the Guide for the Care and Use of Laboratory Animals of the National Institutes of Health. All efforts were made to minimize animal discomfort.

### Restraint stress

Male mice were assigned to either non-stress (NS; n = 15 WT, n = 17 Fgf8 HET) or stress (S; n = 17 WT, n = 14 Fgf8 HET) groups. Each group experienced the exact same testing procedures, except the non-stress mice were left in their cages and the stress group were removed and restrained in ventilated 50 mL conical tubes for 1 h.

### Elevated plus-maze (EPM)

Immediately after 1 h of non-stress or restraint stress, mice were placed on a black acrylic EPM for 5 min. The EPM consisted of a center area (5 cm×5 cm) from which two opposing open arms (29 cm×5 cm) and two opposing closed arms with the same dimensions and walls (15 cm high) were extended. The maze was elevated 60 cm off the ground. Mice were placed in the center area of the EPM facing an open arm to start the test as previously described [25], [26]. Mice that fell off the maze were excluded from analysis (n = 4 WT, n = 2 Fgf8 HET). After the test was completed, mice were returned to their cages. Behavioral testing commenced within 2 h and was completed within 6 h of light phase onset. Room lighting was approximately 480 lx. The EPM was cleaned with 70% ethanol before testing and between each test subject. A video camera was mounted above the EPM to record behavior for later scoring by an observer blinded to the genotypes and groups. The entries or total duration within an area began when all four paws crossed into the area of interest. The time spent in the open, closed and center areas, and number of entries into each arm were scored manually. For analysis, the time spent on the arms and number of entries were expressed as a percentage of the total test duration and number of arm entries, respectively.

### Motor coordination

Baseline balance and motor coordination were tested using an accelerating rotarod (Ugo Basile) in a separate cohort of WT (n = 12) and Fgf8 HET (n = 16) mice. The test consisted of three trials separated by 15 min inter-trial intervals. Four animals were tested together in separate compartments on a rod 3 cm in diameter. Initial velocity was 4 rpm and the rod was gradually accelerated to a maximum of 40 rpm over 5 min. The latency to fall off of the rod during a 5 min test period was recorded. Passive rotations were considered a failure in performance, and the latency to the first full passive rotation was recorded as the latency to fall.

### Tissue collection

Two hours after the onset of the non-stress or stress conditions, mice were deeply anesthetized with isoflurane and decapitated. Brains were removed, and one set was immersion-fixed in 4% paraformaldehyde for 24 h at 4°C then stored in a 30% sucrose cryoprotectant at 4°C, and another set was flash-frozen in isopentane and stored at −70°C until processing for immunohistochemistry (IHC) and high-performance liquid chromatography with electrochemical detection (HPLC-ED), respectively.

### Immunohistochemistry (IHC)

Double immunohistochemical staining for c-Fos (the protein product of the immediate-early gene *c-fos*) and tryptophan hydroxylase (Tph; the rate-limiting enzyme for serotonin biosynthesis) was performed (WT: n = 5 NS, n = 5 S; Fgf8 HET: n = 6 NS, n = 6 S). Before sectioning for IHC, brains were blocked at the caudal border of the mammillary body using a mouse brain matrix (RBM 2000C, ASI Instruments). The tissue block posterior to the mammillary body containing the raphe nuclei was immediately sectioned using a cryostat into 30 µm frozen coronal floating sections that were collected into a series of six microcentrifuge tubes filled with a cryoprotectant (30% sucrose, 30% ethylene glycol, 1% polyvinylpyrolidone in 0.2 M sodium phosphate buffer). For IHC, one third of the sections were taken through a series of rinses and sequential incubations on an orbital shaker using a rabbit anti-c-Fos antibody that has been previously characterized [27], [28] (1∶200, SC-253, Santa Cruz Biotechnology), a biotinylated donkey anti-rabbit secondary antibody (711-065-152, Jackson ImmunoResearch Laboratories), a validated avidin-biotin complex (NeutrAvidin, A2666, Life Technologies; Peroxidase-biotinamidocaproyl conjugate, P-9568, Sigma-Aldrich), and reacted with nickel enhanced 3,3′-diaminobenzidine tetrahydrochloride (DAB; D5637, Sigma-Aldrich) for color detection [23], [29]–[31]. This was immediately followed by a second IHC using a sheep anti-tryptophan hydroxylase antibody that has been previously characterized and shown to bind specifically to both isoforms of Tph [30], [31] (1∶800, T8575, Sigma-Aldrich), a biotinylated donkey anti-sheep secondary antibody (713-065-147, Jackson ImmunoResearch Laboratories), and reacted with DAB for color detection as previously described [23], [29]–[31]. After the color reaction, sections were rinsed, mounted on gelatin-coated slides, dehydrated through increasing concentrations of ethanol (70–100%), cleared in Histo-Clear (National Diagnostics), and coverslipped with Permount (Fisher Scientific).

### Neuronal quantification

**Figure 1:**
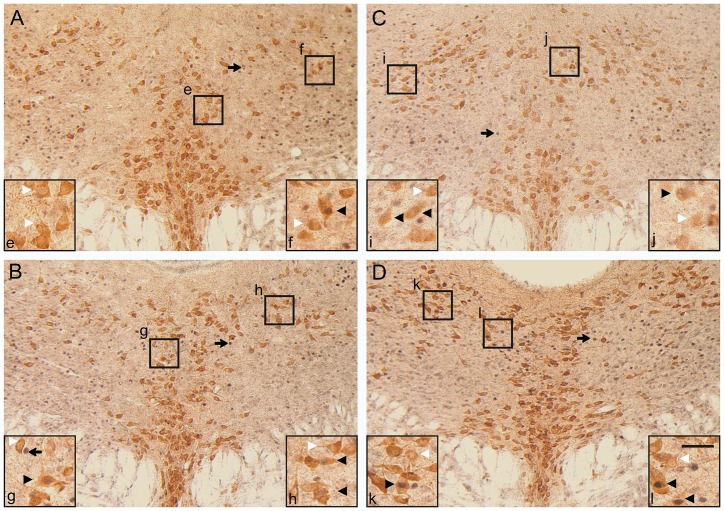
Representative photomicrographs illustrating c-Fos-ir and Tph-ir neurons in the DR at −4.72 mm bregma. WT (A, B) and Fgf8-deficient (C, D) mice exposed to non-stress (A, C) or restraint stress (B, D) conditions. Black boxes with lower case letters, e-l, correspond to higher magnification insets found in the corners of panels A–D. Arrows indicate c-Fos-ir nuclei (blue/black nuclear staining); white arrowheads indicate Tph-ir neurons (brown cytoplasmic staining); black arrowheads indicate c-Fos/Tph-ir double-immunostained neurons. Scale bar represents 100 µm for A–D and 40 µm for insets e–l.

Neurons that were immunoreactive (ir) for c-Fos had a dark blue/black colored nucleus (c-Fos-ir), while Tph-ir neurons were characterized by a brown cytoplasm. Cells that were double-labeled with both c-Fos and Tph and those labeled with only Tph were counted by an investigator blind to the treatment groups at five rostrocaudal levels (−4.36, −4.54, −4.72, −4.90, and −5.08 mm bregma) under a brightfield microscope. Neurons were quantified in the dorsal (DRD; −4.36, −4.54, −4.72, and −4.90 mm bregma), ventral (DRV; −4.36, −4.54, −4.72, and −4.90 mm bregma), ventrolateral part/ventrolateral periaqueductal gray (DRVL/VLPAG; −4.54, −4.72, and −4.90 mm bregma), interfascicular (DRI; −4.72, −4.90, and −5.08 bregma), and caudal (DRC; −5.08 mm bregma) subregions of the DR. For analysis, c-Fos/Tph-ir neurons were expressed as a percentage of the total number of Tph-ir neurons within each subregion at each rostrocaudal level. Representative photomicrographs for each genotype and stress condition at −4.72 mm bregma are shown in Figure 1.

### Brain microdissection and high-performance liquid chromatography with electrochemical detection (HPLC-ED)

To investigate the functional output of DR circuits in WT and Fgf8 HET mice, the concentrations of 5-hydroxyindoleacetic acid (5-HIAA) and serotonin (5-HT) were measured in the DR and select stress-, anxiety-, and panic-related projection sites. Methods for microdissection and HPLC-ED have been previously described [32], [33]. Briefly, brains were cryosectioned into 300 µm-thick slices, placed on slides, and stored at −70°C (WT: n = 5 NS, n = 6 S; Fgf8 HET: n = 9 NS, n = 6 S). The regions selected for microdissection (Table 1) were micropunched under a stereomicroscope using a standard mouse brain stereotaxic atlas as a guide [34]. These regions were chosen because they represent projection sites of anxiety- and stress-related subregions of the DR. Following extraction of micropunched tissues in acetate buffer, 45 µl of the supernatant from each sample was placed in an ESA 542 autosampler (ESA Analytical) and the pellet reconstituted in 0.2 M sodium hydroxide for protein quantification (23235, Thermo Scientific). A total of 25 µl of each sample was injected into the chromatographic system. Chromatographic separation and electrochemical detection were accomplished using a previously described method [13]. Data were normalized by protein content and represented as pg/µg protein. The ratio of 5-HIAA to 5-HT was calculated (5-HIAA/5-HT ratio) and serves as an indication of 5-HT turnover in the DR and projection sites.

**Table 1:** Details of tissue sample collection for HPLC-ED analysis of 5-HIAA and 5-HT concentrations.

Brain region	Rostrocaudal level(mm bregma)	Micropunched sections(number, [diameter µm])
Infralimbic cortex (IL)	1.70, 2.00	4, [410]
Prelimbic cortex (PrL)	1.70, 2.00	4, [410]
Central amygdaloid nucleus (CE)	−1.20, −1.50	4, [410]
Basolateral amygdaloid nucleus (BLA)	−1.50, −1.80, −2.10	6, [410]
Cornu ammonis 1 of dorsal hippocampus (CA1d)	−2.20, −2.50	12, [410]
Cornu ammonis 1 of ventral hippocampus (CA1v)	−3.00, −3.30	12, [410]
Dorsolateral periaqueductal gray (DLPAG)	−4.20, −4.50; −4.80	4, [410]; 2, [310]
Dorsal raphe nucleus, dorsal part (DRD)	−4.20, −4.50, −4.80	3, [310]
Dorsal raphe nucleus, ventral part (DRV)	−4.50, −4.80	2, [310]
Dorsal raphe nucleus, interfascicular part (DRI)	−4.50, −4.80, −5.10	3, [310]
Dorsal raphe nucleus, ventrolateral part/ventrolateralperiaqueductal gray (DRVL/VLPAG)	−4.50, −4.80	4, [410]
Dorsal raphe nucleus, caudal part (DRC)	−5.10	1, [410]

### Corticosterone enzyme-linked immunoassay

To investigate the effects of non-stress or restraint stress conditions on corticosterone levels in WT and Fgf8-deficient mice, a separate cohort of mice (WT: n = 7 NS, n = 7 S; Fgf8 HET: n = 7 NS, n = 7 S) was sacrificed immediately after 1 h of control or restraint stress and their trunk blood collected into heparinized tubes. Plasma samples were isolated by centrifugation and stored at −20°C until the measurement of corticosterone by a commercial enzyme-linked immunoassay kit (ADI-900-097, Enzo Life Sciences) according to manufacturer’s instructions. The intra- and inter-assay coefficients of variation were 6.6−8.4% and 7.8−13.1%, respectively, and the limit of detection was 26.99 pg/ml.

### Statistical analysis

All statistical analyses were completed using SPSS Statistics (version 21.0 for Mac; IBM). All data were analyzed using two-way analysis of variance (ANOVA) for genotype and stress except the rotarod data, which were analyzed using repeated measures ANOVA. Post-hoc analysis was performed using Fisher’s Protected LSD. Data that failed the homoscedasticity test were ln (n+1)-transformed. Statistical outliers were determined using the Grubbs’ test and were removed [35]. For the EPM, 1 out of 57 data points for each the percent time in open, closed and center area were excluded (1.8% of total data for each), and 2 out of 57 data points for the percentage of open entries were excluded (3.5%). For the rotarod, 4 out of 84 data points (4.8%) were excluded. Missing rotarod data were replaced using the Peterson method for the repeated measures ANOVA [36]. For the percent c-Fos/Tph-ir and total number of Tph-ir neurons in each DR subregion, 15 and 5 out of 330 data points each (4.5% and 1.5%, respectively) were excluded. For the concentrations of 5-HIAA and 5-HT, 14 and 12 out of 310 data points each (4.5% and 3.9%, respectively) were excluded. For the 5-HIAA/5-HT ratio 22 out of 310 data points (7.1%) were excluded. For all of the HPLC data combined, 48 out of 930 data points (5.2%) were excluded. There were no outliers for the corticosterone data. Values were shown as the mean ± SEM. Data were considered significant when *p*<0.05.

## Results

### Anxiety-like behavior and motor coordination

**Figure 2:**
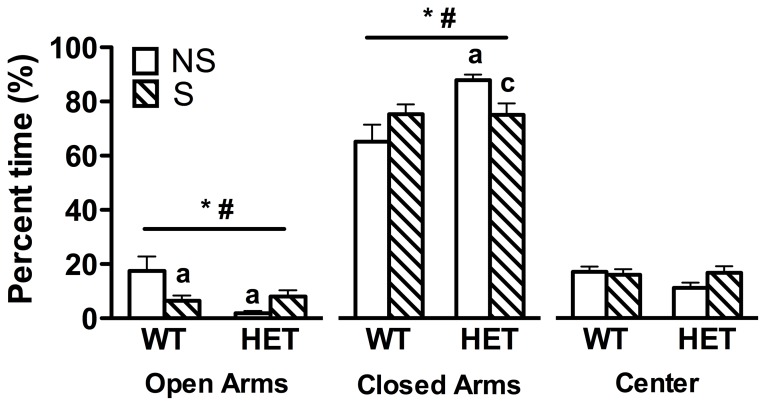
Fgf8-deficient mice have elevated anxiety-like behavior. Anxiety-like behavior was tested using the elevated plus-maze following non-stress (NS) or restraint stress (S) in WT and Fgf8-deficient (HET) mice. Restraint stress significantly increased anxiety-like behavior in WT mice as measured by a lower percentage of time spent in the open arms. Non-stress Fgf8 HET mice spent significantly less time in the open arms and more time in the closed arms than WT non-stress mice. No differences were found for the percent time spent in the center area. N = 12−16/group. Data are presented as mean ± SEM, ^a^p<0.01 vs. WT NS, ^c^p<0.05 vs. HET NS, *main effect of genotype, ^#^genotype x stress interaction.

As shown in Figure 2, there was a significant main effect of genotype on the percentage of time spent in the open and closed arms of the EPM [*F*(1, 52) = 6.3, *p = *0.015, *F*(1, 52) = 7.4, *p = *0.009, respectively]. There was also a significant interaction between genotype and stress on the percentage of open entries, and percent time spent in the open and closed arms of the EPM [*F*(1, 52) = 7.9, *p = *0.007, *F*(1, 52) = 9.4, *p = *0.003, *F*(1, 52) = 7.7, *p = *0.008, respectively]. The mean percentage of open entries were 23±6, 10±2, 8±2, and 16±4 for WT non-stress, WT stress, Fgf8 HET non-stress, and Fgf8 HET stress mice, respectively (mean ± SEM). Post hoc analysis revealed that restraint stress reduced the percentage of open entries (*p = *0.018) and percentage of time WT mice spent on the open arms (*p = *0.007; Figure 2) compared to non-stress WT mice. Similarly, non-stress Fgf8 HET mice had a lower percentage of entries into the open arms (*p = *0.006) and less overall time on the open arms (*p<*0.001; Figure 2) compared to WT non-stress mice. Non-stress Fgf8 HET mice spent a larger percentage of time in the closed arms (*p<*0.001) versus non-stress WT and stress Fgf8 HET mice (*p = *0.031). There were no significant differences between genotypes or stress conditions in the percent time spent in the center area, the total number of closed arm entries (a measure of exploratory behavior and motor function, data not shown), or latency to fall from the rotarod (indicating that motor function was intact in Fgf8 HET mice, data not shown).

### Neuron counts

**Figure 3:**
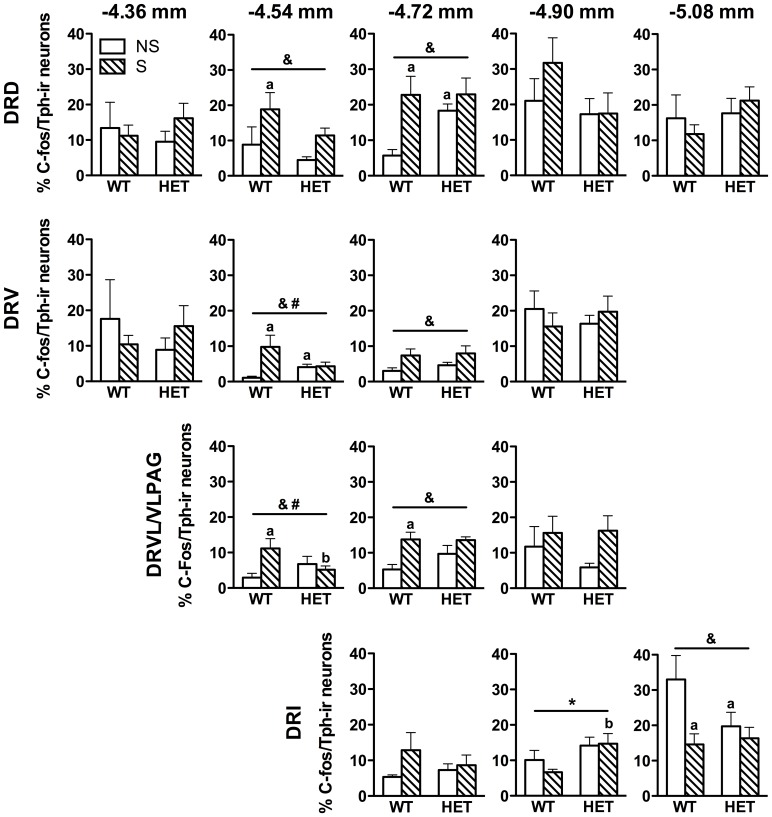
Serotonergic neuronal activation is dysregulated in Fgf8-deficient mice. The percentage of DR c-Fos/Tph-ir neurons at five rostrocaudal levels in WT and Fgf8-deficient (HET) mice following non-stress (NS) or stress (S) conditions. DR subregions are organized by row, and anatomical levels are organized in columns from rostral (left) to caudal (right). Distance from bregma (in mm) is shown above each column. Restraint stress increased serotonergic neuronal activation (% c-Fos/Tph-ir) in the anxiety-related DRD and panic-inhibiting DRVL/VLPAG of WT mice compared to non-stress WT mice. In contrast, activation of serotonergic neurons was similar between the non-stress and stress conditions in Fgf8 HET mice. Non-stress Fgf8 HET mice had increased basal activation of the anxiety-related DRD at −4.72 mm bregma compared to non-stress WT mice and blunted stress-induced activation of the panic-inhibiting DRVL/VLPAG at −4.54 mm bregma compared to WT mice. N = 5−6/group. Data are presented as mean ± SEM, p<0.05, ^a^vs. WT NS, ^b^vs. WT S, ^&^main effect of stress, ^#^genotype x stress interaction. See abbreviations in Table 1.

To investigate the functional integrity of stress-, anxiety-, and panic-related serotonergic circuitries in Fgf8 HET mice, we quantified the percentage of serotonergic cells that co-expressed c-Fos and Tph (c-Fos/Tph-ir) following restraint stress (Figure 3). Two-way ANOVA revealed a significant effect of stress on the percentage of c-Fos/Tph-ir neurons in several mid- and caudal subregions of the DR. These included the DRD, DRVL/VLPAG (Figure 3), and DRV at −4.54 mm and −4.72 mm bregma, and the DRI at −4.90 mm bregma. In general, post hoc analysis revealed that restraint stress increased the percentage of c-Fos/Tph-ir neurons in WT mice (−4.54 mm bregma, DRD *p = *0.03, DRV *p = *0.003, DRVL/VLPAG *p = *0.015; −4.72 mm bregma, DRD *p = *0.01, DRVL/VLPAG *p = *0.008) except in the caudal DRI where stress was associated with a lower percentage of c-Fos/Tph-ir neurons (*p = *0.01). There was a significant genotype x stress interaction in the percentage of c-Fos/Tph-ir neurons in the DRV and DRVL/VLPAG at −4.54 mm bregma. Non-stress Fgf8 HET mice had a higher percentage of c-Fos/Tph-ir than WT non-stress mice in the DRD (*p = *0.041) at −4.72 mm bregma. Fgf8 HET mice also had significantly fewer Tph-ir neurons in the DRV at −4.90 mm bregma compared to WT mice [*F*(1, 17) = 5.8, *p = *0.027; Table A in File S1].

### 5-HIAA tissue content

**Figure 4:**
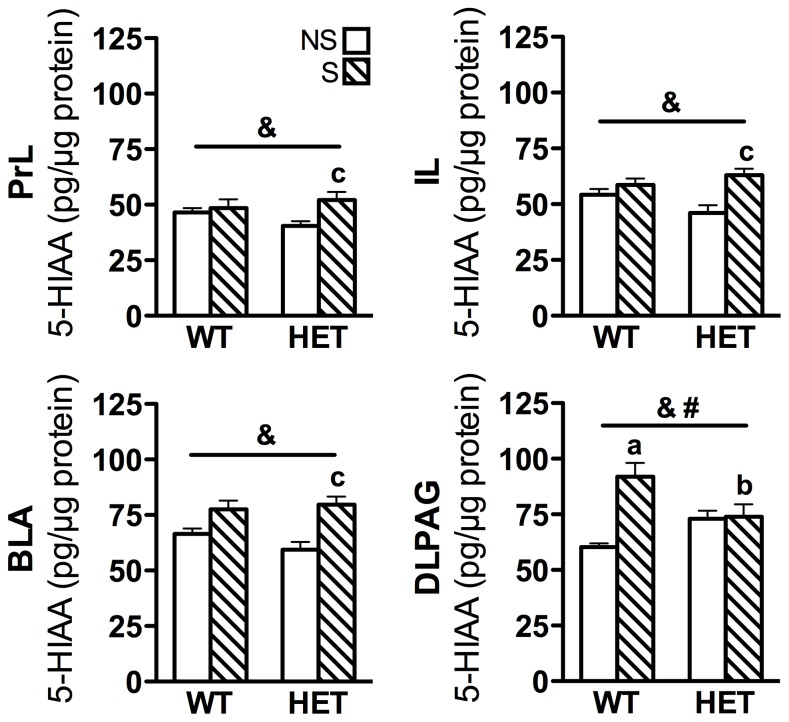
Serotonin metabolism is dysregulated in Fgf8-deficient mice. The 5-HIAA tissue content in anxiety-related and panic-related DR circuits of WT and Fgf8-deficient (HET) mice following non-stress (NS) or restraint stress (S) conditions. Stress increased 5-HIAA content in select anxiety-related projection sites of the DRD (BLA, PrL and IL) as well as in the panic-inhibiting DRVL/VLPAG and DLPAG projection. In WT mice, restraint stress increased 5-HIAA content in the panic-inhibiting DRVL/VLPAG and DLPAG circuit but not in anxiety-related DR projection sites (BLA, PrL, and IL). In Fgf8 HET mice, converse effects were observed. In the DLPAG, Fgf8 HET mice also had lower 5-HIAA content following stress compared to WT mice. N = 4−9/group. Data are presented as mean ± SEM, p<0.05, ^a^vs. WT NS, ^b^vs. WT S, ^c^vs. HET NS, ^&^main effect of stress, ^#^genotype x stress interaction. See abbreviations in Table 1.

To investigate whether Fgf8 deficiency impacted serotonergic functional output in anxiety-, panic- and stress-related circuits, tissue content of 5-HIAA (Figure 4, Table 2), 5-HT (Table B in File S1) and the 5-HIAA/5-HT ratio (Table C in File S1) were analyzed. Overall, stress had similar effects on each of the parameters (5-HIAA, 5-HT, and 5-HIAA/5-HT ratio) in a number of brain regions analyzed. We focus on 5-HIAA in the anxiety-promoting DRD and panic-inhibiting DRVL/VLPAG circuits as the data are consistent with behavioral changes and c-Fos activation, and 5-HIAA was previously shown to be a good indicator of serotonergic metabolism (output) [37]. Two-way ANOVA revealed a significant effect of stress on 5-HIAA content in both the anxiety-promoting DRD and the panic-inhibiting DRVL/VLPAG projection sites [BLA: *F*(1, 22) = 18.0, *p<*0.001, PrL: *F*(1, 20) = 4.6, *p = *0.045, IL: *F*(1, 20) = 9.5, *p = *0.006, DLPAG: *F*(1, 20) = 10.2, *p = *0.005; Figure 4]. There was also a significant genotype x stress interaction in 5-HIAA content in the DLPAG [*F*(1, 20) = 9.2, *p = *0.007]. Post hoc analysis revealed that in the panic-inhibiting DRVL/VLPAG circuit, stress increased both DRVL/VLPAG (Table 2) and DLPAG 5-HIAA content (Figure 4) in WT mice (*p = *0.044 and *p = *0.001, respectively) but only the former in Fgf8-deficient mice (*p = *0.001, Figure 4 and Table 2). In the anxiety-promoting DR projection sites (BLA, PrL, IL), post hoc analysis revealed stress increased 5-HIAA content in Fgf8 HET mice but not WT mice (*p = *0.017, *p = *0.010, and *p = *0.001, respectively, Figure 4).

**Table 2:** 5-HIAA concentrations (pg/µg protein) across brain regions in WT and Fgf8 HET mice following non-stress or stress conditions.

Brain region	5-HIAA (pg/µg protein)								F-statistic, p-value
	WT				HET				
	NS		S		NS		S		
	Mean	SEM	Mean	SEM	Mean	SEM	Mean	SEM	
CE	74	5	83	7	80	2	83	5	n.s.
CA1d	77	5	89	5	65	3	89	6	^&^F(1, 22) = 17, p = 0.001
CA1v	104	11	112	2	102	6	116	5	n.s.
DRD	134	11	164	16	128	6	150	14	^&^F(1, 22) = 4.8, p = 0.04
DRV	179	13	198	12	169	11	195	13	n.s.
DRVL/VLPAG	118	11	163	15	118	9	175	17	^&^F(1, 19) = 14, p = 0.001
DRC	163	9	187	13	144	21	230	34	^&^F(1, 21) = 5.3, p = 0.03
DRI	242	22	264	11	206	8	282	19	^&^F(1, 20) = 12, p = 0.003

Data are presented as mean ± SEM, ^&^main effect of stress, n.s. = not significant. See abbreviations in Table 1.

## Discussion

In this study, we used acute restraint stress and behavioral testing to examine the functionality of the DR serotonergic system in Fgf8-deficient mice. We found both anxiety-promoting and panic-inhibiting serotonergic neurocircuits were altered in these mice, and these changes were associated with elevated baseline anxiety-like behavior in Fgf8-deficient mice. Consistent with previous reports [7], [38]–[40], stress was generally associated with increased DR serotonergic neuronal activation and metabolism in widespread DR projection sites. Differences in DR serotonergic neuronal activation and 5-HT metabolism between genotypes were limited to mid-rostrocaudal to caudal DR subregions and related projection sites. For example, stress increased DRD and DRVL/VLPAG serotonergic neuronal activation in WT mice. Interestingly, similar baseline activation of serotonergic DRD neurons was found in non-stress Fgf8-deficient mice, and their DRVL/VLPAG serotonergic neuronal activation was blunted following stress. This blunted DRVL/VLPAG serotonergic activation was associated with diminished 5-HT metabolism in the DLPAG. Disruption of these subregions and their concomitant connectivity has previously been correlated with chronic anxiety-like states and vulnerability to panic- and anxiety-like behavior [41]. Together these data highlight the importance of developmental Fgf8 signaling in establishing functional anxiety- and panic-related DR serotonergic neurocircuits.

### Elevated anxiety-like behavior in Fgf8-deficient mice

Exposure of WT mice to restraint stress increased anxiety-like behavior as measured by the EPM immediately following stress, whereas anxiety-like behavior was equally elevated in both the non-stress and stress Fgf8-deficient mice. This is consistent with previous studies reporting that stress can precipitate anxiety-like behavior [42], [43] as well our finding that Fgf8 deficiency is associated with increased baseline anxiety-like behavior [23]. These data suggest that Fgf8-deficient mice are more susceptible to stress- and anxiety-related stimuli as they have exaggerated behavioral responses to mild aversive stimuli (such as EPM exposure) compared to WT controls [44]. This anxiogenic phenotype is not related to differences in the magnitude of the neuroendocrine stress response (Text D in File S1), and likely reflects a functional disruption of central anxiety-related circuits resulting from Fgf8 deficiency.

### Altered neuronal activation in anxiety- and panic-related DR subregions of Fgf8-deficient mice

Stress increased c-Fos expression in the mid-rostrocaudal DRD and DRVL/VLPAG serotonergic neurons in WT mice, which is consistent with previous studies examining the effects of stress on the activation of DR serotonergic neurons [7]. In Fgf8-deficient mice, however, there was activation of the DRD in the non-stress group and blunted DRVL/VLPAG activation in response to stress compared to WT mice. The DRD and DRVL/VLPAG have been implicated in responses to stress-, anxiety- and panic-related stimuli, suggesting that these subregions of the DR may be especially sensitive to stress- or anxiogenic challenges [7]. In particular, the DRD has been associated with anxiety-promoting responses while the DRVL/VLPAG is related to inhibiting panic-like responses [14], [45]. In support of this functional assignment, inescapable stress, anxiogenic drugs, fear-potentiated startle, and the avoidance task in the elevated T-maze activate serotonergic DRD neurons [46]–[49], and panicogenic stimuli such as hypercapnia or sodium lactate infusions activate DRVL/VLPAG serotonergic neurons that are associated with inhibition of panic-like behavior [13], [50]. Increased activation of the DRD in non-stress Fgf8-deficient mice compared to WT non-stress mice is consistent with their anxiety-like phenotype. Similarly, blunted activation of DRVL/VLPAG neurons in response to stress is consistent with a failure to activate the panic- and anxiety-suppressing serotonergic system, a circuit normally recruited in response to moderate stressors [14]. Together these data suggest that disruptions in DRD and DRVL/VLPAG neuronal activation may contribute to increased vulnerability to panic- and anxiety-like behaviors in Fgf8-deficient mice.

Activation of serotonergic neurons in the DRD and DRVL/VLPAG following stress is likely related to afferent input from neural circuits regulating stress and emotional responses. DRD afferents include forebrain regions such as the IL, PrL, and bed nucleus of the stria terminalis, and some of these connections are reciprocal. The DRVL/VLPAG receives input from both forebrain and brainstem regions such as the IL, CE, lateral parabrachial nucleus, and the nucleus of the solitary tract (see reviews [7], [51]). The activational perturbations in Fgf8-deficient mice may represent a primary defect in serotonergic neurons (i.e. 5-HT synthesis, firing rate, release, uptake, receptor expression and sensitivity) or may reflect abnormal afferent input as a result of Fgf8 deficiency. There is evidence that Fgf signaling modulates neurite outgrowth and internal cellular calcium concentration following N-methyl-D-aspartate receptor activation [52], [53]. Hence it is possible that loss of Fgf8 signaling impacts the ability of serotonergic neurons to form proper synaptic connections due to abnormal dendritic growth or branching and to respond appropriately to excitatory input. It has also been shown that Fgfs act as target-derived organizers that guide differentiation of both excitatory and inhibitory presynaptic terminals [54]. In this regard, loss of Fgf8 in the developing DR may result in a subregional imbalance of excitatory and inhibitory synapses, and subsequently abnormal responses to stress-related and anxiogenic stimuli.

### Altered 5-HIAA levels in anxiety- and panic- related neural circuits of Fgf8-deficient mice

Fgf8-deficient mice had greater stress-induced increases in 5-HIAA in anxiety-promoting DR target regions as well as decreased 5-HIAA in a panic-suppressing DR target region (Figure 4, Table 2). Together these effects promote an anxiety-like phenotype and may be a consequence of abnormalities within serotonergic neurons, serotonergic DR target regions or with afferent input to the DR due to Fgf8 deficiency. For example, DR has reciprocal connections with the ventral medial prefrontal cortex (vmPFC: IL, PrL) [51]. 5-HT released in the vmPFC can bind postsynaptic 5-HT inhibitory receptors (5-HT_1A_) or excitatory 5-HT_2A_ receptors located on excitatory and inhibitory neurons [55]. The vmPFC in turn sends glutamatergic projections to the DR that largely inhibit serotonergic neurons via activation of local γ-aminobutyric acid synthesizing neurons [56]–[58] or activation of the 5-HT_1A_ autoreceptors due to intra-DR 5-HT release [58]. Fgf8 is expressed in the developing vmPFC and a loss of Fgf signaling in this region results in overall telencephalic hypoplasia, loss of glutamatergic neurons, and axon targeting defects [59]–[62]. These data, combined with the role of Fgfs in neurite outgrowth and synapse formation, raise the possibility that defects in serotonergic projection neurons, vmPFC afferents, or both, may result in elevated stress-induced tissue 5-HIAA content in the vmPFC. Although less is known about the developmental roles of Fgf8 in the DLPAG and BLA, similar mechanisms may account for altered 5-HIAA content in those regions. Additional studies are needed to explore these possibilities and whether Fgf8-related structural abnormalities in other brain regions contribute to vulnerability to stress- and anxiety-related behaviors.

Of note, Fgf8-deficient stress mice have reduced DRVL/VLPAG serotonergic neuron activation which is mirrored by reduced 5-HIAA content in the DLPAG. However, the relationship between serotonergic neuron activation and 5-HIAA content in the DRD and the projection regions (PrL, IL, and BLA) is less clear. 5-HIAA content in these DRD projection sites may not exactly mirror changes in DRD serotonergic activation because these projection sites receive serotonergic input from numerous sources, including the DRI and median raphe nucleus (MnR) [7]. Additionally, the DRD may have inhibitory connections with the DRI/MnR [63]. Loss of DRD inhibition of the DRI/MnR would then disinhibit these serotonergic neurons to increase 5-HIAA content. This additional input may dissociate the activation level of DRD serotonergic neurons with 5-HIAA content in DRD projection regions.

One caveat about this study is that all animals, stress or non-stress, have been exposed to EPM. EPM alone may affect the outcome of neurochemical tests in non-stress animals and could also interact with restraint stress in stressed animals. For example, one study has found that exposure of rats to the avoidance, but not the escape, component of the elevated T-maze increased c-Fos expression in DRD serotonergic neurons while having no effect on DRVL/VLPAG serotonergic neurons [49]. These findings raise the possibility that EPM exposure in the stress Fgf8-deficient mice, but not WT, was sufficient to increase c-Fos expression in DRD serotonergic neurons. We acknowledge this confound, but it does not invalidate our observation that Fgf8-deficient mice respond differently from WT mice under both stress and non-stress conditions. Further, an important question in the present study is whether behavioral changes parallel neurochemical changes. This question necessitates that behavioral and neurochemical tests be conducted on the same animals.

In conclusion, this study demonstrates that reduced Fgf8 signaling is correlated with increased anxiety-like behavior and dysregulated serotonergic neuron activation and functional output in response to stress- and anxiogenic stimuli. The functional disruption is particularly prominent in DRD and DRVL/VLPAG serotonergic neurocircuits. Our results provide strong support for an early role of Fgf8 signaling in programming stress- and anxiety-related serotonergic neurocircuits responsible for proper behavioral response to stress. Specifically, these data suggest that Fgf8 signaling is not only critical for the early formation and positioning of DR neurons but also the integration of serotonergic neurons into functional stress- and anxiety-related circuitries.

## Supporting Information

File S1 Contains the following supporting information files: **Table A.** Number of Tph-ir neurons for each subregion of the DR at different rostrocaudal levels in WT and Fgf8 HET mice. **Table B.** 5-HT concentrations (pg/µg protein) across brain regions in WT and Fgf8 HET mice following non-stress or stress conditions. **Table C.** 5-HIAA/5-HT ratios across brain regions in WT and Fgf8 HET mice following non-stress or stress conditions. **Text D.** Plasma corticosterone.
(DOCX)
