# Supplementary material for: Fibroblast Growth Factor 8 Deficiency Compromises the Functional Response of the Serotonergic System to Stress
Source: PLoS One. 2014 Jul 3;9(7):e101420. doi: 10.1371/journal.pone.0101420 (PMC4081718; doi:10.1371/journal.pone.0101420)
Supplement: File S1 — Contains the following supporting information files: Table A. Number of Tph-ir neurons for each subregion of the DR at different rostrocaudal levels in WT and Fgf8 HET mice. Table B. 5-HT concentrations (pg/µg protein) across brain regions in WT and Fgf8 HET mice following non-stress or stress conditions. Table C. 5-HIAA/5-HT ratios across brain regions in WT and Fgf8 HET mice following non-stress or stress conditions. Text D. Plasma corticosterone. (DOCX) [file pone.0101420.s001.docx]

**Supporting information**

**Table A.** Number of Tph-ir neurons for each subregion of the DR at different rostrocaudal levels in WT and Fgf8 HET mice

| Subregion | Rostrocaudal level  (mm bregma) | WT | | HET | |
| --- | --- | --- | --- | --- | --- |
|  |  | Mean  (#) | SEM | Mean (#) | SEM |
| DRD | −4.36 | 78 | 11 | 82 | 9 |
| DRV | −4.36 | 47 | 6 | 66 | 11 |
| DRD | −4.54 | 103 | 8 | 93 | 10 |
| DRV | −4.54 | 134 | 11 | 147 | 11 |
| DRVL/VLPAG | −4.54 | 125 | 21 | 125 | 17 |
| DRD | −4.72 | 67 | 9 | 65 | 8 |
| DRV | −4.72 | 111 | 11 | 107 | 9 |
| DRVL/VLPAG | −4.72 | 121 | 17 | 137 | 17 |
| DRI | −4.72 | 49 | 4 | 48 | 5 |
| DRD | −4.90 | 50 | 3 | 49 | 4 |
| DRV | −4.90 | 56 | 7 | 39* | 2 |
| DRVL/VLPAG | −4.90 | 41 | 7 | 47 | 6 |
| DRI | −4.90 | 75 | 7 | 73 | 6 |
| DRC | −5.08 | 61 | 9 | 55 | 7 |
| DRI | −5.08 | 62 | 6 | 60 | 5 |

Non-stress and stress groups were combined for analysis. Data are presented as mean ± SEM, *p< 0.05 vs. WT. See abbreviations in Table 1.

**Table B.** 5-HT concentrations (pg/μg protein) across brain regions in WT and Fgf8 HET mice following non-stress or stress conditions

| Brain region | 5-HT (pg/μg protein) | | | | | | | | F-statistic, p-value |
| --- | --- | --- | --- | --- | --- | --- | --- | --- | --- |
|  | WT | | | | HET | | | |  |
|  | NS | | S | | NS | | S | |  |
|  | Mean | SEM | Mean | SEM | Mean | SEM | Mean | SEM |  |
| BLA | 16.1 | 0.78 | 17.1 | 2.1 | 14.6 | 0.41 | 18.0 | 1.7 | n.s. |
| CE | 16.9 | 1.6 | 14.4 | 1.7 | 18.6 | 0.92 | 18.8 | 2.2 | n.s. |
| CA1d | 9.9 | 0.10 | 10.0 | 0.44 | 9.7 | 0.66 | 10.1 | 0.41 | n.s. |
| CA1v | 17.5 | 1.9 | 17.1 | 0.29 | 18.5 | 0.87 | 18.7 | 1.5 | n.s. |
| PrL | 7.3 | 1.4 | 5.8 | 0.66 | 6.7 | 0.29 | 6.8 | 0.71 | n.s. |
| IL | 9.6 | 0.45 | 7.6 | 0.64 | 8.5 | 0.39 | 9.2 | 0.56 | ^#^F(1, 19) = 6.5, p = 0.020 |
| DLPAG | 13.9 | 1.2 | 19.2 | 1.1 | 20.1 | 1.5 | 17.0 | 1.3 | ^#^F(1, 22) = 8.4, p = 0.008 |
| DRD | 54.2 | 5.0 | 65.9 | 5.2 | 57.5 | 3.2 | 62.7 | 5.9 | n.s. |
| DRV | 69.9 | 5.5 | 69.2 | 6.9 | 73.6 | 4.0 | 77.2 | 3.1 | n.s. |
| DRVL/VLPAG | 33.1 | 3.1 | 47.9 | 5.5 | 37.7 | 3.0 | 52.1 | 6.4 | ^&^F(1, 19) = 9.0, p = 0.007 |
| DRC | 47.7 | 4.3 | 49.8 | 4.6 | 52.1 | 6.6 | 62.2 | 6.5 | n.s. |
| DRI | 65.7 | 7.2 | 53.1 | 6.5 | 63.0 | 2.4 | 59.5 | 6.4 | n.s. |

Data are presented as mean ± SEM, ^&^main effect of stress, ^#^genotype x stress interaction, n.s. = not significant. See abbreviations in Table 1.

**Table C.** 5-HIAA/5-HT ratios across brain regions in WT and Fgf8 HET mice following non-stress or stress conditions

| Brain  Region | 5-HIAA/5-HT ratio | | | | | | | | F statistic, p-value |
| --- | --- | --- | --- | --- | --- | --- | --- | --- | --- |
|  | WT | | | | HET | | | |  |
|  | NS | | S | | NS | | S | |  |
|  | Mean | SEM | Mean | SEM | Mean | SEM | Mean | SEM |  |
| BLA | 4.2 | 0.50 | 4.3 | 0.22 | 4.1 | 0.26 | 4.6 | 0.50 | n.s. |
| CE | 4.5 | 0.25 | 5.9 | 0.40 | 4.3 | 0.14 | 5.5 | 0.60 | ^&^F(1, 19) = 15.3, p = 0.001 |
| CA1d | 7.7 | 0.55 | 8.9 | 0.43 | 6.9 | 0.37 | 8.8 | 0.36 | ^&^F(1, 22) = 13.1, p = 0.002 |
| CA1v | 6.0 | 0.37 | 6.5 | 0.08 | 5.6 | 0.49 | 5.8 | 0.08 | n.s. |
| PrL | 7.0 | 1.1 | 8.6 | 0.78 | 6.1 | 0.32 | 7.7 | 0.63 | ^&^F(1, 20) = 6.4, p = 0.020 |
| IL | 5.7 | 0.24 | 7.8 | 0.51 | 5.5 | 0.24 | 6.9 | 0.31 | ^&^F(1, 19) = 24.1, p < 0.001 |
| DLPAG | 4.8 | 0.33 | 4.8 | 0.09 | 3.9 | 0.24 | 4.4 | 0.10 | *F(1, 20) = 9.2, p = 0.007 |
| DRD | 2.5 | 0.11 | 2.5 | 0.11 | 2.3 | 0.11 | 2.4 | 0.14 | n.s. |
| DRV | 2.6 | 0.12 | 2.9 | 0.17 | 2.3 | 0.08 | 2.7 | 0.19 | ^&^F(1, 21) = 7.8, p = 0.011 |
| DRVL/VLPAG | 3.6 | 0.07 | 3.5 | 0.08 | 3.2 | 0.13 | 3.5 | 0.21 | n.s. |
| DRC | 3.5 | 0.15 | 3.9 | 0.42 | 3.5 | 0.43 | 3.3 | 0.18 | n.s. |
| DRI | 3.8 | 0.23 | 5.2 | 0.39 | 3.3 | 0.07 | 4.5 | 0.54 | ^&^F(1, 20) = 19.7, p < 0.001 |

Data are presented as mean ± SEM, ^&^main effect of stress, *main effect of genotype, n.s. = not significant. See abbreviations in Table 1.

**Text D**. *Plasma corticosterone*

Compared to non-stress animals, plasma corticosterone (ng/mL) was significantly elevated in the stress group in both genotypes [*F*(1, 24) = 108.9, *p <* 0.001, mean ± SEM, WT: 138 ± 11 NS; 383 ± 27 S; Fgf8 HET: 142 ± 16 NS; 361 ± 29 S]. There was no main effect of genotype or a genotype x stress interaction.

**Abbreviations**

BLA, basolateral nucleus of the amygdala; CA1d, CA1 region of the dorsal hippocampus; CA1v, CA1 region of the ventral hippocampus; CeA, central nucleus of the amygdala; EPM, elevated plus-maze; DLPAG, dorsolateral periaqueductal gray; DRC, dorsal raphe nucleus, caudal part; DRD, dorsal raphe nucleus, dorsal part DRI, dorsal raphe nucleus, interfascicular part; DRV, dorsal raphe nucleus, ventral part; DRVL/VLPAG, dorsal raphe nucleus, ventrolateral part/ventrolateral periaqueductal gray; Fgf, fibroblast growth factor; IL, infralimbic cortex; MnR, median raphe nucleus; PrL, prelimbic cortex; vmPFC, ventral medial prefrontal cortex; 5-HT, 5-hydroxytryptamine (serotonin); 5-HIAA, 5-hydroxyindoleacetic acid
